# Pharmacist—Pharmacy Technician Intraprofessional Collaboration and Workplace Integration: Implications for Educators

**DOI:** 10.3390/pharmacy8020095

**Published:** 2020-06-01

**Authors:** Maryam Jetha, Ali Walji, Paul Gregory, Dalya Abdulla, Zubin Austin

**Affiliations:** 1 Leslie Dan Faculty of Pharmacy, University of Toronto, Toronto, ON M5S 3M2, Canada; maryam.jetha.16@ucl.ac.uk (M.J.); ali.walji16@ucl.ac.uk (A.W.); streetsoccercanada@mac.com (P.G.); 2 Faculty of Applied Health and Community Studies, Sheridan College Institute of Technology and Advanced Learning, Brampton, ON L6Y 5H9, Canada; dalya.abdulla@sheridancollege.ca; 3 Leslie Dan Faculty of Pharmacy and the Institute for Health Policy, Management, and Evaluation—Faculty of Medicine, University of Toronto, Toronto, ON M5S 3M2, Canada

**Keywords:** pharmacy technician, collaboration, community pharmacy practice, pharmacy technician education, continuing professional development

## Abstract

Globally, concerns have been expressed regarding the impact of regulation of pharmacy technicians. After more than a decade of experience with technician regulation in Ontario, Canada, uptake of the full scope of practice for technicians has been sporadic at best. The objective of this study was to examine barriers and facilitators to intraprofessional collaboration between pharmacists and pharmacy technicians for the purpose of identifying possible curricular or educational interventions to enhance workplace integration. A qualitative, interview-based study of 24 pharmacists, technicians, educators, pharmacy managers, and owners was undertaken using a semi-structured interview guide. Key findings of this research include: (i) Confirmation of suboptimal utilization of regulated technicians in practice; (ii) identification of crucial knowledge and skills gaps for both pharmacists and technicians; and (iii) proposals for undergraduate education and training, and continuing professional development learning opportunities to address these gaps. In order to achieve the promise and potential of regulation of pharmacy technicians, system-wide change management—beginning with education—will be required and will benefit from multiple stakeholder engagement and involvement.

## 1. Introduction

Historically, the role of the pharmacy technician in Ontario, Canada, has grown out of the work of those who assisted pharmacists in day-to-day dispensary related duties [1]. As an unregulated workforce under direct supervision and control of pharmacists, pharmacy assistants performed a wide variety of activities, including dispensing, compounding, inventory control, and in some cases, provision of education, support, or information to patients. In this role, assistants had little or no formal legal liability, no professional responsibilities, and unstandardized education, training, and practice-readiness assessment [2,3]. It is difficult to pinpoint when and the reasons why the assistant role evolved into the (unregulated) pharmacy technician role, and why the change in title occurred. Formal education, assessment, and clinical training programs for pharmacy technicians began to proliferate in the 1980s and 1990s, usually in second-order post-secondary educational institutions (e.g., community colleges rather than universities) [1,3,4]. Over time, standardized curricula and assessment models evolved, as did a growing professional ethos that viewed the pharmacy technician role as complementary to, rather than completely subsumed under, the role of the pharmacist [2]. As the technical complexity of pharmacy work evolved, thus too has the job description, expectations, and academic requirements for pharmacy technicians. In many jurisdictions—including Ontario—pharmacy technicians and pharmacy assistants work side by side, potentially leading to confusion for both the public and pharmacists who may not understand the distinction between the two roles and titles [3,4,5]. 

The regulation of pharmacy technicians was initially proposed and enacted as part of a broader strategy to support pharmacists in providing an expanded scope of practice and more impactful patient care services to the public [5,6]. A recent study suggested that regulated pharmacy technicians could yield time savings of close to 20%, allowing pharmacists greater opportunities for patient care [6]. Other studies suggest that the quality of pharmacy technician-led dispensing was higher, with lower error rates and greater operational efficiency [7]. Regulators viewed regulation as an important step in enhancing standardization of education and training, ensuring minimal competency, and providing a formal vehicle for defining responsibility and legal liability for the work undertaken by pharmacy technicians [8]. In regulating pharmacy technicians, there was also an implicit understanding that a professional ethos would grow, and standards of practice, competency expectations, and continuing professional development requirements would evolve [9,10]. Many jurisdictions in Europe, the UK, the US, and Canada began to develop formal frameworks for the regulation of pharmacy technicians while still maintaining an informal and unregulated pharmacy assistant role [11,12].

Ontario is the largest province in Canada; at present, there are 4861 registered pharmacy technicians [1]. The Ontario College of Pharmacists (the regulatory body for the profession) has implemented a regulatory scheme for pharmacy technicians that provides opportunities for pharmacists to focus on cognitive services while providing a high degree of independence for technicians to perform a variety of other important dispensary-related activities (see Table A1) [1,10]. It was hoped that this scheme would promote greater intraprofessional collaboration, facilitating expanded opportunities for pharmacists to engage in cognitive services aimed at enhancing patient care outcomes. Anecdotally, this hope has not been fully realized, particularly in the community pharmacy sector [10]. Regulated technicians continue to be underutilized with respect to their knowledge and skills, and pharmacists are unclear as to how best to leverage this workforce to open up additional opportunities for non-technical patient care services [13]. Worse, regulated technicians have noted that, despite additional educational requirements and regulatory obligations (both of which are costly and time-consuming), they have not enjoyed enhanced professional, employment, or remuneration opportunities commensurate with their increasingly professionalized role [13]. While regulated pharmacy technicians in the hospital sector appear somewhat better integrated with the intraprofessional workflow, community-based regulated pharmacy technicians appear to be less impactful—and satisfied—than was initially hoped [10,14,15].

Recently, there has been increased interest in exploring barriers and enablers to the integration of regulated technicians in the workforce. Renfro et al. examined employer perceptions of pharmacy technicians in community settings and highlighted knowledge and skills gaps related to interpersonal competencies that may limit fuller integration [16]. Banks et al. have examined the economic benefits of pharmacy technicians practicing at advanced scope; they similarly note that further development of the technician curriculum may be required in order to truly unleash the potential of the role, though they do not provide specific recommendations for curricular content reform [17]. Desselle has used an organizational behavior framework to describe pharmacy technician work life. This work highlighted the need for self-actualization and the quest to provide value to the organization as important issues and signpost ways in which current education and training programs serve as barriers and facilitators to fuller integration [11]. Across much of this literature, there is little explicit focus on pharmacy technician curriculum and training as an object of research interest.

The objective of this research was to understand and describe barriers and facilitators to intraprofessional collaboration and integration between community-based regulated pharmacy technicians and pharmacists, with a particular emphasis on educational and curricular gaps in knowledge and skills.

## 2. Materials and Methods 

As there has been little formal research exploring the issue of integration and collaboration between pharmacists and regulated pharmacy technicians, exploratory research was used in order to explore the boundaries of this research while providing opportunities to define future areas for focused research. A qualitative research method was selected in order to support the integration of diverse opinions and experiences and to ensure multiple stakeholders were involved and engaged in the research process. Semi-structured interviews were identified as the main data-gathering tool for this research; one-on-one interviews with multiple stakeholders would allow for the fullest exploration of different experiences and beliefs in a manner that would encourage individual participants to be honest and forthright in their disclosures. Since the emphasis in this project was on implications for educators, 3 major cohorts were identified for inclusion as participants: Regulated pharmacy technicians (both senior-level students and recently qualified individuals), community pharmacists (both practitioners and managers/owners), and educators (in both pharmacy technician and pharmacy programs). While each cohort would bring different perspectives, there would also be significant overlap between categories; for example, some participants would be both pharmacy owners and pharmacy educators. The term “educator” in this study was applied broadly and included those who delivered formal planned lectures within a post-secondary education program, as well as those who served as clinical mentors/preceptors to trainees (either pharmacy technician or pharmacy students) within their community practices, and for whom teaching was not a primary professional identity. Given the exploratory nature of this research, we felt it was important to support a broad array of different participants with diverse experiences and backgrounds, all focused on the same topic of interest; to that end, the same semi-structured interview guide (see Table A2) was used for all participants—regardless of background—though latitude was built into this protocol to support the participant’s emphasizing areas or topics of specific personal interest.

A combination of convenience, snowballing, and purposive sampling was used to recruit participants. Convenience sampling involved research team members approaching known informants with specific expertise or interest in this research area with an invitation to participate. Snowball sampling involved asking these participants to recommend other colleagues who they felt might be interested in participating and who may be valuable in helping the research team address our objectives. Purposive sampling involved a deliberate attempt to ensure that convenience and snowball sampling methods did not inadvertently result in excess of certain categories of participants (e.g., too many regulated technicians or too few pharmacy educators). Importantly, no attempt was made to ensure the demographic representativeness of participants. Initial recruitment for this study was undertaken through a general announcement placed on social media (Facebook and Instagram), indicating the purpose of the study and inviting individuals who were interested in participating to self-identify. Participants were then informally approached by members of the research team to consider involvement in a 30–45-min audiotaped interview to discuss issues related to pharmacist-pharmacy technician integration and collaboration. If agreeable, participants completed informed consent pursuant to a research protocol approved by the University of Toronto. Participants were informed that no compensation was available for involvement in this study. A research team member would then arrange to interview the participant at a mutually convenient time in person, by phone, or through a mutually agreed-upon technology platform (such as skype, GoogleHangouts, Facebook Messenger, etc.). In addition to audio and/or video recording of interviews, researchers maintained field notes to help confirm understanding of content. 

Verbatim transcripts of all interviews were produced and analyzed using an inductive coding method described by Yin [18]. The trustworthiness of data interpretation was established through triangulation principles described by Lincoln and Guba [19]: Each transcript was reviewed by a minimum of 2 independent researchers who developed, then reconciled coding structures to categorize data and develop themes. Field notes were used to confirm thematic understanding, and the coding structure evolved iteratively with subsequent analyses. According to the research protocol, interviews were to progress to data saturation, the point at which no additional new information or themes were identified. In practice (given the inevitable lag time between interview, transcription, individual coding, and team reconciliation of themes), more interviews than were necessary were undertaken beyond the point of saturation, and these additional interviews were then available for use for confirmatory and triangulation purposes. All data for this study were managed and maintained using a combination of nVivo 11.1, Microsoft Excel, and Microsoft Word.

## 3. Findings and Discussion

A total of 24 individuals from different backgrounds participated in this research; see Table A3 for the demographic profile of participants. As noted, there were 9 recent pharmacy technician graduates, 9 students currently enrolled in pharmacy technician education programs, and 6 were community pharmacists who also participated (to varying degrees and intensity) as pharmacy technician or pharmacy educators (either as formal lecturers in academic programs or as clinical mentors/preceptors for experiential training).

Based on this research, three key themes were identified: (a) Integration of regulated pharmacy technicians in community pharmacy is neither consistent nor is it widespread; (b) fuller integration is limited due to identified knowledge and skills gaps that potentially could be addressed through educational programs for pharmacists and pharmacy technicians; and (c) lack of a clear business model to ensure the sustainability of an integrated workforce is a major obstacle to fuller integration.

(a) Integration of regulated pharmacy technicians in community pharmacy is neither consistent nor is it widespread

Across all interviews and participants, a clear and common perspective was the “disappointing” way in which the integration of regulated pharmacy technicians has progressed in community practice in Ontario.

*“Well you can’t help but feel a bit depressed by it. I mean in school they tell us that we are going to be regulated and professionals and this will open up so many opportunities and jobs and like we are really needed and everything. But then when you start working—well, it was exactly the same as before I went to school, I got a little bit of a raise but really nothing, and the job was still the same.”*

Most participants in this research suggested that a career pathway for regulated technicians was clearer and better established within hospital pharmacies rather than in community practice, in part because the work of pharmacists and technicians is more clearly delineated.

*“Of course it works better in hospital because everyone—well, there is a specific job for the [regulated] technician and the pharmacist doesn’t do that anymore.”*

Similar to findings by Alkhateeb et al [20], most of the pharmacy technicians (students, recent graduates, and teachers) also noted that larger organizations, some of which are unionized, create better working conditions for a fuller expression of the regulated pharmacy technician roles and responsibilities.

*“I guess it makes sense. I mean a typical drug store, they don’t have the resources so of course there are limits. I think it’s also that in hospital some of the [regulated technicians] are unionized, right, so the unions, well they fight for the rights of the technicians to make sure they are respected and all that.”*

A key finding of this study was confirmation that, despite over a decade of legislation and support, integration of regulated pharmacy technicians in community practice has been sporadic at best, and most regulated technicians are not practicing to their fullest potential or scope.

(b) Fuller integration is limited due to identified knowledge and skills gaps that could potentially be addressed through educational programs for pharmacists and pharmacy technicians.

Participants in this study described the integration of regulated technicians into community practice as a process that appears to have become stalled or incomplete. When asked to reflect upon causes, all participants highlighted gaps in knowledge and skill that limited capacity for integration. Key gaps included: (i) Communication/interpersonal skills; (ii) conflict management and negotiation skills; (iii) professionalism and professional ethics; and (iv) practice management/practice readiness. Importantly, these gaps were almost entirely and consistently framed around so-called “soft-skills” deficits, rather than foundational knowledge (e.g., pharmacology, jurisprudence), or technical/procedural skills (e.g., dispensing, compounding, bulk manufacturing, etc.). Though describing the same or substantially similar gaps, pharmacists in this study framed the issue as one of “trusting” regulated technicians with certain tasks, while technicians framed the same issue as one of “self-confidence” in performing tasks.

*“I say this as someone who is both a manager of a pharmacy and who teaches in [a technician education program]. Even some of my best students…well, you have to have faith, you have to trust them to do certain things on their own, without you there and I’m not sure most of them…well, the program just doesn’t provide them with that level of preparation to deal with real world issues and patients.”*

(i) Key communication/interpersonal gaps related to the basic understanding of patient psychology, motivational interviewing, and human behavior, as opposed to basic customer service skills. Understanding of issues such as the psychology of health and illness, complex family dynamics, managing cultural diversity, and cultural competency, and dealing with diverse sexualities and orientations were all identified as crucial for success in community practice but frequently absent in the formal education, training, and assessment of regulated technicians. Most participants described the need for nuanced and sophisticated interpersonal skills in community pharmacy practice as an essential factor for success; pharmacists, technicians, students and educators all agreed that pharmacy technician education programs do not adequately prepare students for this reality and that in most cases students selecting these programs do not have significant natural strengths in these areas:

*“Working with patients—it’s really hard. And I think most pharmacy technicians, I mean the kind of person that studies this—well, they likely are more on the technical side not the personal side so this won’t be something that comes naturally or easily to them, working with difficult situations and patients. That’s why the programs—the teachers—really need to step up and make sure this is part of the program itself, to teach people to get them ready for reality. Otherwise no, integration will never happen, it can’t happen”*.

(ii) Conflict Management and Negotiation Skills
  One particular sub-type of communication skills was strongly emphasized as a knowledge and skill gap by all participants in this research—the ability to manage conflict and successfully negotiate outcomes with diverse patients and colleagues. Conflict management consistently emerged as one of the most important skills deficits that limited fuller integration of pharmacy technicians into community practice, and again was framed as a “Trust” issue by pharmacists and a “self-confidence” issue by technicians. As noted by Pervanas et al. [21]. This is particularly important in the context of communication across dispensing errors or near misses. Most participants expressed the belief that conflict management and negotiation are skills that can be taught and assessed, and strongly endorsed the idea that this should be integral to the technician education program and curriculum, on part with basic courses such as pharmacology or pharmacy math:

*“It’s the most common thing in [community pharmacy] right? You’re always getting into disagreements or arguments – about insurance, drug shortages, whatever. And I know for myself, my nature—I’m just not the kind of person that knows how to argue properly or to stand up for myself or just stay calm when things get heated. It’s really frustrating—I mean, I don’t think I actually SHOULD be trusted with some of the jobs regulated technicians are eligible to do…I know I don’t have the skills you need so I’m glad the pharmacist is there to do it instead. I just wish though they had taught me more about this in school.”*

(iii) Professionalism and Professional Ethics
  The question of whether pharmacy technicians are “professionals” is one that has parallels for pharmacists; the technical focus of the field itself may suggest they are not professionals, while the responsibilities and unique skills associated with the role are highly suggestive of profession-hood. Participants in this study noted challenges they experienced in articulating what professional status means in the context of pharmacy technicians and tended to revert to a series of behavioral characteristics (e.g., punctuality, reliability, honesty) rather than character traits or occupational characteristics associated with specific and unique knowledge and skills. Further to this point, most participants in this study noted that the current structure of community pharmacy practice limited the formation of a professional identity as pharmacists maintained significant control over most activities. This inhibition of professional identity formation was seen as significantly detrimental to fuller integration in practice.

“You know, I find it ironic I guess. For years I’ve heard pharmacists complain about doctors holding them back, putting them down, not letting them do their best or their jobs to the fullest, and how this wastes [the pharmacist’s] talent and potential as a professional. That’s exactly what pharmacists are doing to [pharmacy technicians]. They don’t let us flourish, and then they complain we’re not professional enough or integrating well enough!”

For many technicians in this study, a large part of this problem relates to the lack of a truly independent scope of practice unique from pharmacists; so long as pharmacists can (legally) do everything regulated technicians can do, there would be no need for a separate profession. In the absence of a unique skill set and body of knowledge that does not duplicate what pharmacists already do, no unique professional ethics can—or needs to—evolve, and this further stunts the development of the field into a profession and limits true integration.

“Let’s be real…at the end of the day it still all comes down to what the pharmacist wants and does and as long as that’s the case, we’ll always only be helpers, not actual professionals. If there were actually real decisions I had to make on my own, and take responsibility—then yeah I’d be a professional. But I’m not. What frustrates me though is that a lot of times I know these pharmacists—well besides a degree there’s nothing special about them that is different from most [regulated pharmacy technicians] but still, that’s the way it is.”

Most participants in this study identified the need for and value of focused education and training in the area of professionalism and professional ethics as a way of enhancing both self-confidence and trustworthiness in pharmacy technicians to assume more complex layers of responsibility and thereby integrate more fully into practice. The notion that professionalism and professional ethics are things that can be taught, learned, and assessed—and should be within the pharmacy technician education program—was widely endorsed by all participants in this study and highlighted as a specific strategy to enhance better uptake of technicians in community practice.

(iv) Practice Management/Practice Readiness
  A final category of knowledge and skills gaps identified by participants in this study that limited full integration in community practice were practice management and practice readiness capabilities. These capabilities were broadly framed as a series of attributes related to self-motivation, ability to balance risks and benefits, capacity to make and carry out decisions in information-imperfect environments, and ability to take on and assume responsibility rather than simply defer to another authority. Such attributes are essential to smooth day to day management and functioning of a community pharmacy, but are rarely explicitly addressed, taught, or assessed in pharmacy technician education programs.

*“I guess the biggest issue I have with trusting [regulated pharmacy technicians] to do more is the sense that most of them—well that I know at least—well, they are a bit passive. They don’t seem to want to take on responsibility to manage their own problems but instead are always asking for permission, instead of showing initiative. I get that of course—but if they don’t have confidence in their own ability, why should I have confidence in their abilities? So it ends up that they end up being stuck in low level positions, and then complain that we aren’t letting them live up to their potential.”*

The circularity in the logic of the comment above highlights a dilemma that was described by many participants, a push-pull between pharmacists and technicians with respect to how much independent responsibility is expected, allowed, or tolerated. Without opportunities to test-drive independent responsibility, these skills cannot develop or flourish—but without self-motivation and initiative to drive the process forward, such opportunities are rarely freely given. The extent to which these are issues of personality and character vs. issues of skills training and curriculum development are unclear; nonetheless, many participants noted how important these capabilities are for successful workplace integration and endorsed the notion that these need to be explicitly included within pharmacy technician education programs. Desselle et al. [22] highlighted similar issues in their examination of future trends in pharmacy technician education. Participants in this study confirmed this perspective and signposted future opportunities for greater partnerships between pharmacy technician and pharmacist education programs, as well as greater alignment within workplaces and with professional associations to take a more coordinated approach to workforce integration.

(c) Lack of a clear business model to ensure the sustainability of an integrated workforce is a major obstacle to fuller integration.

The final common theme through this research was related to the lack of a viable, sustainable business model for integrating higher-paid regulated pharmacy technicians into practice. Commensurate with their knowledge, skills, training, qualifications, and advanced credential, there is a general expectation that regulated pharmacy technicians should be remunerated at a higher level than regulated pharmacy assistants. As this research highlighted, the lack of a clear role for technicians in practice connects to a lack of a viable business plan related to higher rates of remuneration:

“It’s a chicken and egg thing…the [regulated technicians] think they deserve to be paid more—higher—than the pharmacy assistants. That’s fair, makes sense. But where does that money come from? What are they doing to add sufficient value and generate additional revenue to make this possible? That’s not clear yet. It’s not up to [regulated pharmacy technicians] to figure this out…it’s up to pharmacy owners and businesses to create the business plan that makes this possible, and so far – we haven’t. So that means integration doesn’t happen as well as it could because no one can figure out how to pay for it. Maybe this is something that pharmacy researchers should be working on to help the profession deal with this.”

A business plan that simultaneously appropriately compensates regulated pharmacy technicians, creates additional value in the business to warrant and support this increased compensation, and is both viable and sustainable appears difficult to develop given the current financial climate in community pharmacy. While the initial premise of regulation of technicians focused on the notion that regulated technicians would take over technical dispensary duties, freeing up time for pharmacists to undertake more remunerative cognitive service activities such as medication reviews or immunization consultations, the economics of this business case has not been sustainable or viable given significant reductions in income sources from cognitive services for community pharmacy. As a result, no participant in this study was able to identify a best-practice model for sustainable, viable, economically feasible integration of regulated pharmacy technicians in the workplace. Further research into this theme is required to better understand economic barriers and facilitators that were described as important by participants. 

## 4. Strengths and Limitations

This study is unique in attempting to explore the issue of pharmacy technician workplace integration from the perspective of curriculum and training, using the qualitative method focused on multiple-stakeholder perspectives. The research method used triangulatory processes designed to confirm themes across multiple stakeholders; all transcripts were read and assessed independently by two coders who achieved consensus on code definitions and theme descriptions, further enhancing the trustworthiness of analysis/interpretation.

There are, however, limitations to this research. The qualitative method used and the narrow geographical focus of this study limit generalizability outside of the specific context of Ontario, Canada. While the study method involved interviews until thematic saturation, it is difficult to know if a different cohort of participants would have identified other factors not identified in this research. Findings were based on a participant pool of 24—while this is a reasonably robust number in terms of qualitative studies [9], these participants were not demographically representative, and were identified through purposive, convenience, and snowball sampling methods, which further limits the generalizability of findings. The inclusion of more community pharmacists and community pharmacists with no connection to formal education or clinical training of pharmacy or pharmacy technician students may have also yielded additional or different data for analysis and could be considered in the future. However, as a first step to address a complex issue of topical importance across different jurisdictions, this research has signposted some valuable areas for future exploration and research and has been valuable in highlighting opportunities for evidence-based quality improvement in education and practice.

## 5. Conclusions

The integration of regulated pharmacy technicians in community pharmacy practice continues to be a vexing issue in many different jurisdictions. This study has helped identify potential areas for curricular quality improvement in pharmacy technician education programs that may enhance the quality and extent of integration. Most of these areas fall into a broad category of “soft-skills” training needs; further work is required to verify the results of this study and determine the feasibility of teaching, learning, and assessment of these soft skills as a method for enhancing the impact that regulated pharmacy technicians may have on community practice and their patients. Particular areas for curricular attention include interpersonal communication skills appropriate for clinical/care workplaces, conflict management and negotiation, intraprofessional collaboration skills, and practice management competencies. There may also be opportunities to consider joint pharmacy/pharmacy technician student education workshops or events in order to start to build a more intraprofessionally collaborative culture at the student level. Another important insight from this study is the need for further work to identify financially viable and sustainable practice models that integrate regulated technicians into the workforce. In recognition of their advanced education, qualifications, and scope of practice, it is reasonable that regulated pharmacy technicians expect and deserve a wage premium compared to unregulated technicians: Understanding how to accommodate this wage premium within an existing business and financial structure, and leveraging the advanced education, qualifications, and scope of practice to generate additional revenue to support payment of higher wages has not been clearly determined within most practices in this study, and this further limits workplace integration. Educators and researchers have an important potential role in working with individual practices and the profession as a whole to provide business plan templates that can support employers in making decisions to hire and retain regulated technicians in a fiscally responsible and sustainable manner. 

In a recent study by Anderson et al. [23], the lack of standardization in pharmacy technician education and training programs was identified as a potential barrier to fuller utilization of technicians and integration in practice. Similarly, Wheeler et al. have noted that “(w)ith ongoing pharmacist practice transformation, an approach that ensures uniform technician education…is vital to support a practice model designed to transform medication management across the continuum of care [24]”. This research has potentially contributed valuable information regarding specific areas for focus in enhancing the quality, rigor, and impact of the educational programs, which form the foundation of regulated pharmacy technician practice.

## Appendix A

**Table A1:** Allocation of task responsibilities in community pharmacy.

Pharmacy Task	Who Can Perform
Input a Prescription	Pharmacy Assistant, Pharmacy Technician, Pharmacist
Prepare a Prescription	Pharmacy Assistant, Pharmacy Technician, Pharmacist
Final Check of Product Preparation	Pharmacy Technician, Pharmacist
Check of Clinical Appropriateness	Pharmacist
Patient Consultation	Pharmacist

**Table A2:** Semi-structured interview protocol.

**Opening Questions**
1. Tell me a little about your background, i.e.,
• What steps did you take/currently taking to become a Pharmacist/Technician/Assistant (location, education, college/school)?
• Why did you decide to pursue this path?
• How long have you been practicing?

2. What employment experience have you had?
• Name of Pharmacy? Urban, suburban or rural area?
• What was the daily prescription volume of the store?
• Who does the pharmacy team in your store consist of on a typical shift? (i.e., ratio of pharmacists, pharmacy technicians, students, pharmacy assistants)
NB: If no pharmacy technician hired—ask why this is the case

**Theme 1: Work environment**
1. Would you mind explaining to me the pharmacy workflow and staff roles every time a new prescription arrives? How do you work as a team?
Educators only: What do you understand are areas where your students struggle with the most in the workplace? How do you feel these can be addressed?
2. On the first day, how are you provided information about your own role and those of other staff?
3. In your experience, what worked well to optimise your role in the pharmacy?
a. Probe: Any workplace practices, policies, training, physical design of pharmacy, i.e., designated workstations, workflow?
4. In your experience, what areas could be improved to better facilitate team work?
a. Probe: Any workplace practices, policies, training, physical design of pharmacy, i.e., designated workstations, workflow?
**Theme 2: Scope of practice**
1. How do you feel you/pharmacy technician makes a difference to the pharmacy?
2. What do you know about your scope/ the scope of practice of pharmacy technicians? Where did you learn about it?
Educators only: Do you feel the students have a complete understanding of your scope of practice as a pharmacy technician student? How do they learn this?
3. What do you feel differentiates a pharmacist from a regulated pharmacy technician?
4. What do you feel differentiates a pharmacy technician from a pharmacy assistant?
**Theme 3: Education**
1. How effectively did your school curriculum prepare you for real world work and what specifically was in that curriculum?
2. What do you learn at school about workplace integration and delegating or communicating effectively as part of a team? What could be improved?
Educators only: What do students learn at college about workplace integration, delegating or communicating effectively as part of a team?
3. How would you like this information to be provided if at all and what would be the ideal package to help you successfully integrate?
**Theme 4: Professional Identity & Confidence in the workplace**
1. How confident (if you were to rate out of ten) are you in interacting with the following groups?
a. Interacting with patients
b. Interacting with licensed Pharmacy Technicians
c. Interacting with Pharmacy Assistants
d. Interacting with Student Pharmacists
e. Interacting with registered Pharmacists
f. Answering phone calls from other health care practitioners
Educators only: In the workplace, how confident do your graduate pharmacy technicians feel interacting with their community pharmacy team and patients following the course? What has been successful and what may be improved?
2. If you were to speak with your pharmacy team, what would you like to tell them about how you would like to be worked with?
3. In the workplace, do you feel that pharmacy technicians are utilised to their full extent?
4. In your experience, do you feel that enough trust is placed on a pharmacy technician to carry out delegated roles?

**Closing questions**
1. How do you feel about the future of your career?
2. How do you see your role changing in the next 5 years?
3. You may have alluded to this already, but finally what advice would you give to pharmacists and employers to optimise the role of the regulated pharmacy technician?
4. Any other thoughts or opinions?

**Table A3:** Demographic Profile of Research Participants (n = 24).

Code	Sex	Age *	Roles	Background
RPT1F30	F	30	Recent Pharm Tech Graduate, employed	Worked as assistant × 5 years prior to enrolling in program
RPT2F28	F	28	Recent Pharm Tech Graduate, employed	Supervises bulk packaging and compounding
RPT3F35	F	35	Pharm Tech Graduate, employed	Works in 3 different community practices
RPT4F48	F	48	Recent Pharm Tech Graduate, employed	Former pharmacist from another country, requalified as technician
RPT5M29	M	29	Recent Pharm Tech Graduate, employed	Worked as assistant × 2 years prior to enrolling in program
RPT6M44	M	44	Recent Pharm Tech Graduate, unemployed	Former pharmacist from another country, requalified as technician, currently attempting licensure as pharmacist
RPT7F49	F	49	Recent Pharm Tech Graduate, employed	Worked as assistant × 22 years prior to enrolling in program
RPT8F22	F	22	Recent Pharm Tech Graduate, employed	Worked as student assistant × 4 years prior to enrolling in program
RPT9F29	F	29	Recent Pharm Tech Graduate, unemployed	Parental leave
PTS1F40	F	40	Pharm Tech Student	Worked as assistant × 20 years prior to enrolling in program
PTS2F57	F	57	Pharm Tech Student	Former pharmacist from another country, attempting to qualify
PTS3F22	F	22	Pharm Tech Student	No previous experience
PTS4F28	F	28	Pharm Tech Student	No previous experience
PTS5F33	F	33	Pharm Tech Student	Worked as assistant × 9 years prior to enrolling in program
PTS6M48	M	48	Pharm Tech Student	Former pharmacist from another country, attempting to qualify
PTS7M42	M	42	Pharm Tech Student	Former pharmacist from another country attempting to qualify
PTS8M30	M	30	Pharm Tech Student	No previous experience
PTS9M22	M	22	Pharm Tech Student	No previous experience
RPH1F52	F	52	Pharmacist, pharmacy owner, and educator	Pharmacist × 30 years, educator × 22 years
RPH2F49	F	49	Pharmacist and educator	Pharmacist × 27 years, educator × 11 years
RPH3M50	M	50	Pharmacist, pharmacy owner, and educator	Pharmacist × 25 years, educator × 12 years
RPH4M46	M	46	Pharmacist, pharmacy owner, and educator	Pharmacist × 20 years, educator × 6 years
PTE1F59	F	59	Pharmacist and pharmacy technician educator	Pharmacist × 32 years, educator × 18 years
PTE2M48	M	48	Pharmacist, pharmacy technician educator, pharmacy manager	Pharmacist × 26 years, educator × 12 years

*: if disclosed by participant.
